# Study of Endothelial Dysfunction in Patients With Non-alcoholic Fatty Liver Disease

**DOI:** 10.7759/cureus.20515

**Published:** 2021-12-19

**Authors:** Nijin Jose, Vasant P.K, Kiran G Kulirankal

**Affiliations:** 1 Internal Medicine, Jubilee Mission Medical College and Research Institute, Thrissur, IND; 2 Internal Medicine, Amrita Institute of Medical Sciences, Kochi, IND

**Keywords:** flow-mediated vasodilation, bmi, non-alcoholic fatty liver disease, endothelial dysfunction, metabolic syndrome

## Abstract

Background

Metabolic syndrome (syndrome X) is the name for a group of risk factors that raises your risk for heart disease and other health problems, such as diabetes and stroke. Dilatation of blood vessels following stress is a function of vasodilators produced by the endothelium. Flow-mediated vasodilation assesses endothelial function. In the case of endothelial dysfunction, flow-mediated vasodilation is impaired, resulting in decreased or even absence of vasodilation following stress. The easy availability of ultrasound machines nowadays and the non-invasive nature of the test make this a practical test for assessing endothelial dysfunction and the risk of cardiovascular diseases. Various studies have confirmed the presence of impaired flow-mediated vasodilation in patients with coronary artery disease. However, the presence of impaired flow-mediated vasodilation in individuals with risk factors but no cardiovascular diseases can prove that this can be used to predict individuals at risk. This study tries to confirm the presence of endothelial dysfunction in patients with non-alcoholic fatty liver disease (NAFLD) attending a tertiary center hospital in Kochi.

Objectives

The study's main aim is to compare flow-mediated dilatation in patients with NAFLD and normal individuals.

Materials and methods

The comparative study was conducted among 50 patients attending various outpatient departments in Amrita Institute of Medical Sciences, Kochi. History and examination of cases and controls and relevant investigations were done after obtaining consent. In addition, both groups underwent measurement of flow-mediated vasodilation in the radiology department. Data were entered in Microsoft Excel and were analyzed using SPSS.

Results

Flow-mediated vasodilation was found to be less in patients with fatty liver (7.37 ± 2.75) when compared to individuals with normal liver (12.41 ± 3.71). In addition, flow-mediated vasodilatation was inversely proportional to BMI and age.

Conclusion

This study has proved that there will be endothelial dysfunction in NAFLD, as shown by the decrease in flow-mediated vasodilation when compared with normal liver.

## Introduction

Metabolic syndrome, also known as syndrome X, insulin resistance syndrome, obesity dyslipidemia syndrome, and the deadly quartet, is a cluster of conditions that occur together, increasing the risk of heart diseases, stroke, and type 2 diabetes. Abdominal obesity is associated with insulin resistance leading to hyperinsulinemia, hyperglycemia, and increased adipokine secretion [1, 2]. Complications of insulin resistance include endothelial dysfunction, systemic hypertension, hyperlipidemia, and vascular inflammation [3]. These effects are found even in patients with normal BMI and increased abdominal fat [4]. Genetic susceptibility, lack of exercise, and body fat distribution are the major contributing factors. Syndrome X includes glucose of more than 100 mg/dL or on drug treatment for increased blood sugars, high-density lipoprotein (HDL) less than 40 mg/dL for men and less than 50 mg/dL for women, or on treatment for decreased HDL, triglyceride levels more than 150 mg/dL or on drug treatment for increased triglycerides, a waist size of more than 102 cm in men and 88 cm in women, and blood pressure of more than 135/80 mm Hg or on drug treatment for high blood pressure. At least 3 out of 5 risk factors are needed to satisfy the criteria. Usually, there is more than one component present in an individual. Metabolic syndrome is associated with a prothrombotic and proinflammatory state [3]. It is characterized by an increase in C-reactive protein [5] and interleukin [6]. The incidence of metabolic syndrome varies with age and peak incidence occurs at 60 years. The incidence in the Indian population is around 31.6% [7]. There is an increase in the prevalence of metabolic syndrome mainly due to change in lifestyle and food habits [8]. The main risk factors for the development of metabolic syndrome are obesity, increasing age, postmenopausal state, smoking, high-carbohydrate diet, physical inactivity, and family history of metabolic syndrome [9]. Complications related to metabolic syndrome include the development of type 2 diabetes mellitus [10], cardiovascular diseases [11], non-alcoholic fatty liver disease (NAFLD), hepatocellular carcinoma [12], intrahepatic cholangiocarcinoma, renal dysfunction, polycystic ovarian syndrome, hyperuricemia, and vascular dementia.

NAFLD is characterized by fat accumulation in the liver without any other cause. NAFLD is the most common chronic liver disease in many parts of the world, including India. The incidence of NAFLD in India was estimated to be 9-32% among the general population according to a study done in 2013 [13]. The association between non-alcoholic fatty liver disease and insulin resistance is very strong. The spectrum of the non-alcoholic fatty liver includes hepatic steatosis, non-alcoholic steatohepatitis, cirrhosis, and hepatocellular carcinoma. Of this, hepatic steatosis is the most benign disorder. The factors that affect prognosis include age between 40 and 45 years, obesity, presence of type 2 diabetes mellitus, and genetic susceptibility. Free fatty acids, diacylglycerols, and toxic intermediates like reactive oxygen species are responsible for hepatocyte damage and death. Most of the patients will be asymptomatic, and laboratory investigations will have only a mild elevation of aspartate aminotransferase, alanine aminotransferase, and alkaline phosphatase, making it easy to be overlooked by the clinician and by the patient.

Endothelial function can be assessed via invasive tests such as intracoronary administration of vasoactive substances and non-invasively through the assessment of flow-mediated vasodilation, myocardial positron emission tomography, and peripheral arterial tonometry. Flow-mediated vasodilation assesses endothelial function [14]. Dilatation of blood vessels following stress is a function of vasodilators produced by the endothelium, predominantly nitric oxide. In the case of endothelial dysfunction, this is impaired, resulting in decreased or even absence of vasodilation following stress. This dilation can be easily assessed using ultrasound with a vascular probe. The easy availability of ultrasound machines nowadays and the non-invasive nature of the test make it a practical test for assessing endothelial dysfunction and the risk of cardiovascular diseases. Studies have confirmed the presence of impaired flow-mediated vasodilation in patients with coronary artery disease. The presence of impaired flow-mediated vasodilation in individuals with risk factors but no cardiovascular diseases, can prove that this can be used to predict individuals at risk.

Only a few studies from India have reported impairment of flow-mediated vasodilation in patients with NAFLD without any history of diabetes mellitus, systemic hypertension, smoking, or history of coronary artery disease. Therefore, this study tries to confirm the presence of endothelial dysfunction in NAFLD patients attending a tertiary center hospital in Kochi. Thus, proving that NAFLD is an independent risk factor for the development of coronary artery disease.

## Materials and methods

The study is a comparative study between two groups of patients attending various outpatient departments in Amrita Institute of Medical Sciences, Kochi, conducted over three years.

Patients with NAFLD who are of age more than 18 years of age were included in the study. Patients with diabetes mellitus, systemic hypertension, dyslipidemia, smoking, history of alcoholic intake, coronary artery disease, cerebrovascular disease, chronic kidney disease, sepsis, and serious infections were excluded from the study. Cases are patients with NAFLD in ultrasound abdomen, without other risk factors for endothelial dysfunction (normal liver function test [LFT], renal function tests [RFT], fasting blood sugar [FBS]/Postprandial blood sugar [PPBS]/Hemoglobin A1C [HbA1C], normal blood pressure, Hepatitis B, Hepatitis C serology) and controls will be patients with normal liver in ultrasound without any risk factors for endothelial dysfunction.

Patients with diabetes mellitus, systemic hypertension, dyslipidemia, history of smoking and alcohol intake, coronary artery disease, cerebrovascular disease, chronic kidney disease or sepsis, and serious infections were excluded.

History and examination of cases and controls, along with relevant investigations such as complete blood count, RFT, LFT, fasting lipid profile, FBS/PPBS/HbA1C, hepatitis B, and hepatitis C serology, were done. With the help of the results, the study population was selected. Ultrasound abdomen was used to identify individuals with fatty liver and normal liver. Both groups underwent measurement of flow-mediated vasodilation in the department of radiology, using an ultrasound machine.

Steps for assessing flow-mediated vasodilatation:

1) Baseline value (D1): Brachial artery diameter measured using vascular probe of USG machine, 7.5 cm above medial epicondyle.

2) Sphygmomanometer is tied to the forearm and inflated to a pressure of 250 mm Hg, for a period of five minutes and then pressure is released.

3) Brachial artery diameter is measured at 7.5 cm above medial epicondyle, after 60 s of deflation (D2).

Flow mediated vasodilation = (D2-D1)/D1 x 100

Flow-mediated vasodilation (expressed in %) between two groups was compared.

Statistical analysis was performed using IBM SPSS version 20.0 software.

The age of 25 and gender-matched individuals were selected as cases (fatty liver) and controls (normal) after obtaining informed consent.

## Results

A total of 22 out of 50 individuals were female, who were distributed equally between the normal liver group and fatty liver group. Of the 28 males, 14 were in the normal liver group, and 14 were in the fatty liver group (Table 1).

**Table 1:** Comparison of gender in fatty liver group and normal liver group.

Gender	Group		p-value
	Normal	Fatty Liver	
Female (n = 22)	11 (50)	11 (50)	_
Male (n = 28)	14 (50)	14 (50)	

The mean age of cases was 45.6 ± 18.5, while the mean age in controls was 41.12 ± 14.33, which does not show any statistical significance. In addition, a total of 40% of individuals in controls were doing regular exercise, while only 16% in cases were doing regular exercise. However, the difference was not statistically significant (Table 2).

**Table 2:** Comparison of age in normal group and fatty liver group.

Group	Mean Age	p-value
Normal	41.12±14.33	0.345
Fatty Liver	45.60±18.5	

Flow-mediated vasodilation was found less in patients with fatty liver when compared to individuals with normal liver. Flow-mediated vasodilation in cases (fatty liver group) was found to be 7.37 ± 2.75, and in controls (normal liver group) was 12.41 ± 3.71, which shows statistical significance with p-value <0.001 (Table 3).

**Table 3:** Flow-mediated vasodilation in fatty liver group and normal group.

Group	n	Mean Flow-mediated Vasodilation	SD	p-value
Normal liver group	25	12.4160	3.71682	<0.001
Fatty liver group	25	7.3700	2.75068	

Out of 16 patients with normal BMI, 81.3% of individuals had normal liver, which is not statistically significant. However, 64.7% of individuals with BMI >22.9 had fatty liver, which is statistically significant (Table 4).

**Table 4:** Comparison of BMI in normal liver and fatty liver group.

BMI Category	Group		p-value
	Normal	Fatty liver	
<22.9 (n = 16)	13 (81.3%)	3 (18.8%)	0.006
>22.9 (n = 34)	12 (35.3%)	22 (64.7%)	

The variation in flow-mediated vasodilation with relationship to age and BMI was assessed in two groups separately. In the normal liver group, there is an inverse relationship between age and flow-mediated vasodilation, the r-value is -0.111 but it is not statistically significant. In the same way, as age increases, the flow-mediated vasodilation decreases in the fatty liver group (r-value = - 0.684); this relationship is statistically significant. It is also found that as the BMI increases, the flow-mediated vasodilation decreases in the normal liver group (r-value = -0.135), but it was not statistically significant. However, BMI in the fatty liver group also showed the same inverse relationship (r-value = -0.432), which is statistically significant (Table 5).

**Table 5:** Relationship of age and BMI in flow-mediated vasodilation in normal and fatty liver group.

Group	Variables	Percentage of Vasodilation		
		n	r-value	p-value
Normal	Age	25	-0.111	0.598
	BMI	25	-0.135	0.521
Fatty liver	Age	25	-0.684	<0.001
	BMI	25	-0.432	0.031

It was also found that as age increases, flow-mediated vasodilation decreases (r value= -0.377). This relationship was statistically significant (Table 6).

**Table 6:** Relationship between age and flow-mediated vasodilation in the whole population.

Age	Percentage of Vasodilation		
	n	r-value	p-value
	50	-0.377	0.007

## Discussion

Metabolic syndrome is an ever-growing problem due to lifestyle changes and food habits. As shown by various studies, the incidence has increased considerably in the last decades. Even younger individuals are getting affected by this disease primarily due to lack of physical activity and increased calorie intake due to changes in food habits. Complications of metabolic syndrome include type 2 diabetes mellitus, cardiovascular diseases, increase in all-cause mortality, liver disease, hepatocellular carcinoma, intrahepatic cholangiocarcinoma, renal dysfunction, microalbuminuria, polycystic ovarian syndrome, sleep apnea, hyperuricemia, gout, and vascular dementia. Endothelial function can be assessed by evaluating the vessel’s response to shear stress or vasoactive agents. 

NAFLD is characterized by fat accumulation in the liver without any other cause. It is the most common chronic liver disease in many parts of the world. The prevalence of NAFLD in India was estimated to be 9-32% among the general population according to a study done in 2013. Two clinical features represent NAFLD: non-alcoholic fatty liver (namely “steatosis”), and non-alcoholic steatohepatitis (namely: “steatohepatitis). It has been postulated that endothelial dysfunction could be one of the earliest factors associated with fat accumulation in NAFLD. It has also been widely associated with cardiometabolic syndrome and its components: hepatic and systemic insulin resistance (IR), dyslipidemia, visceral obesity, hypertension, impaired fasting glucose, and increased stroke risk. Insulin resistance per se is responsible for endothelial dysfunction via the imbalance of the enzymatic system of nitric oxide production (eNOS). When insulin resistance appears, it also leads to endothelial dysfunction through the impairment of nitric oxide production and the inhibition of insulin-induced vasorelaxation; eNOS function impairment has been widely associated with it. Moreover, it was demonstrated that endothelial dysfunction with impaired nitric oxide production is involved in the progression of advanced liver diseases such as cirrhosis. In addition, it is associated with increased vascular resistance (resulting in portal hypertension) and hepatic stellate activation in the liver (resulting in fibrosis).

Endothelial function can be easily studied by brachial flow-mediated dilation (FMD) measurement. Flow-mediated vasodilation is calculated as the percentage increase in diameter from baseline to the maximum value, which is obtained after the cuff deflation. A decrease in flow-mediated vasodilation is associated with atherosclerosis of the blood vessels. It improves with risk reduction measures. 

A total of 22 out of 50 individuals were female, who were distributed equally between the normal liver group and fatty liver group. Of the 28 males, 14 were in the normal liver group and 14 were in the fatty liver group. The mean age of cases was 45.6 ± 18.5, while the mean age in controls was 41.12 ± 14.33, which does not show any statistical significance. A total of 40% of individuals in controls were doing regular exercise, while only 16% in cases were doing regular exercise. However, the difference was not statistically significant. Flow-mediated vasodilation was found less in patients with fatty liver when compared to individuals with normal liver. Flow-mediated vasodilation in cases (fatty liver group) was found to be 7.37 ± 2.75 and in controls (normal liver group) was 12.41 ± 3.71, which shows statistical significance with p-value <0.001. This is similar to the study done by Fan Y et al. [15]. Out of 16 patients with normal BMI, 81.3% of individuals had normal liver, which is not statistically significant. However, 64.7% of individuals with BMI >22.9 had fatty liver, which is statistically significant. The variation in flow-mediated vasodilation with relationship to age and BMI was assessed in two groups separately. It was found that in the normal liver group, there is an inverse relationship between age and flow-mediated vasodilation (r-value is -0.111) but it is not statistically significant. In the same way, as age increases, the flow-mediated vasodilation decreases in the fatty liver group (r-value = - 0.684); this relationship is statistically significant. It is also found that as the BMI increases, the flow-mediated vasodilation decreases in the normal liver group (r-value = -0.135), but it was not statistically significant. However, BMI in the fatty liver group also showed the same inverse relationship (r-value = -0.432), which is statistically significant. It was also found that as age increases, flow-mediated vasodilation decreases (r value= -0.377). This relationship was statistically significant. 

This study has proved that there will be endothelial dysfunction, as shown by the decrease in flow-mediated vasodilation, in patients with NAFLD. NAFLD, now considered a part of metabolic syndrome, will increase the cardiovascular risk even in the absence of traditionally considered risk factors like systemic hypertension, diabetes mellitus, dyslipidemia, or smoking. Identifying such patients and starting them on preventive measures will reduce morbidity and mortality. This can be used effectively even in low-cost settings as it is a non-invasive bedside procedure. Emphasis should be stressed on prevention rather than treatment after developing complications.

The study had selected individuals who were attending the outpatient departments in our hospital and may not represent the community. The study did not assess the association between severity of fatty liver and degree of reduction in flow-mediated vasodilation. Patients with diabetes mellitus, systemic hypertension, dyslipidemia, history of smoking and alcohol intake, coronary artery disease, cerebrovascular disease, chronic kidney disease, sepsis, and serious infections were not included in the study and hence will not represent the entire community. The Diet of the patients was also not analyzed in this study. These are some of the limitations of the study.

## Conclusions

The study proves that there is endothelial dysfunction in NAFLD as shown by the decrease in flow-mediated vasodilation when compared with normal liver. Thus, it shows that NAFLD can now be considered a part of metabolic syndrome, increasing an individual's cardiovascular risk even in the absence of traditionally considered risk factors like systemic hypertension and diabetes mellitus, dyslipidemia, or smoking. The study showed that patients with a BMI of more than 22.9 kg/m2 had a higher prevalence of NAFLD than patients with a BMI <22.9 kg/m2. There is an inverse relationship between BMI and flow-mediated vasodilation, as BMI increases, flow-mediated vasodilation decreases. There is an inverse relationship between flow-mediated vasodilation and age, as age increases, flow-mediated vasodilation decreases.
